# Serum high mobility group box 1 as a potential biomarker for the progression of kidney disease in patients with type 2 diabetes

**DOI:** 10.3389/fimmu.2024.1334109

**Published:** 2024-02-28

**Authors:** Tongtong Liu, Hailing Zhao, Ying Wang, Peng Qu, Yanmei Wang, Xiai Wu, Tingting Zhao, Liping Yang, Huimin Mao, Liang Peng, Yongli Zhan, Ping Li

**Affiliations:** ^1^ Guang’anmen Hospital, China Academy of Chinese Medical Sciences, Beijing, China; ^2^ China-Japan Friendship Hospital, Institute of Medical Science, Beijing, China; ^3^ Dongzhimen Hospital, Beijing University of Chinese Medicine, Beijing, China

**Keywords:** HMGB1, biomarker, diabetic kidney disease, progression, T2DM

## Abstract

**Background:**

As a damage-associated molecular pattern protein, high mobility group box 1 (HMGB1) is associated with kidney and systemic inflammation. The predictive and therapeutic value of HMGB1 as a biomarker has been confirmed in various diseases. However, its value in diabetic kidney disease (DKD) remains unclear. Therefore, this study aimed to investigate the correlation between serum and urine HMGB1 levels and DKD progression.

**Methods:**

We recruited 196 patients with type 2 diabetes mellitus (T2DM), including 109 with DKD and 87 T2DM patients without DKD. Additionally, 60 healthy participants without T2DM were also recruited as controls. Serum and urine samples were collected for HMGB1 analysis. Simultaneously, tumor necrosis factor receptor superfamily member 1A (TNFR-1) in serum and kidney injury molecule (KIM-1) in urine samples were evaluated for comparison.

**Results:**

Serum and urine HMGB1 levels were significantly higher in patients with DKD than in patients with T2DM and healthy controls. Additionally, serum HMGB1 levels significantly and positively correlated with serum TNFR-1 (*R* ^2^ = 0.567, *p*<0.001) and urine KIM-1 levels (*R* ^2^ = 0.440, *p*<0.001), and urine HMGB1 has a similar correlation. In the population with T2DM, the risk of DKD progression increased with an increase in serum HMGB1 levels. Multivariate logistic regression analysis showed that elevated serum HMGB1 level was an independent risk factor for renal function progression in patients with DKD, and regression analysis did not change in the model corrected for multiple variables. The restricted cubic spline depicted a nonlinear relationship between serum HMGB1 and renal function progression in patients with DKD (*p*-nonlinear=0.007, *p*<0.001), and this positive effect remained consistent across subgroups.

**Conclusion:**

Serum HMGB1 was significantly correlated with DKD and disease severity. When the HMGB1 level was ≥27 ng/ml, the risk of renal progression increased sharply, indicating that serum HMGB1 can be used as a potential biomarker for the diagnosis of DKD progression.

## 1 Introduction

Diabetes mellitus is a major cause of chronic kidney disease (CKD) worldwide and is a strong risk factor for the progression of CKD to end-stage renal disease (ESRD) (1, 2). Diabetic kidney disease (DKD) affects up to 40% of patients with diabetes and is associated with a substantial incidence and mortality of ESRD and cardiovascular events (3, 4). The mortality risk associated with early-stage DKD is much higher than that associated with CKD or diabetes without DKD (5). The estimated glomerular filtration rate (eGFR) and albuminuria are established markers for assessing renal function progression (6, 7). However, there is a significant difference between albuminuria and renal impairment in diabetic neuropathy, and DKD can occur without increased albuminuria and subsequent progression to ESKD (8, 9). On the other hand, owing to the regulation of complex environmental and genetic factors, there is heterogeneity in the progression rate of CKD in patients with diabetes, which is manifested by the rapid decrease in the eGFR in some populations with DKD. Conversely, others experience a more indolent course (10, 11). Therefore, it is necessary to identify new biomarkers that can reliably predict the development and progression of DKD.

**Figure 1:**
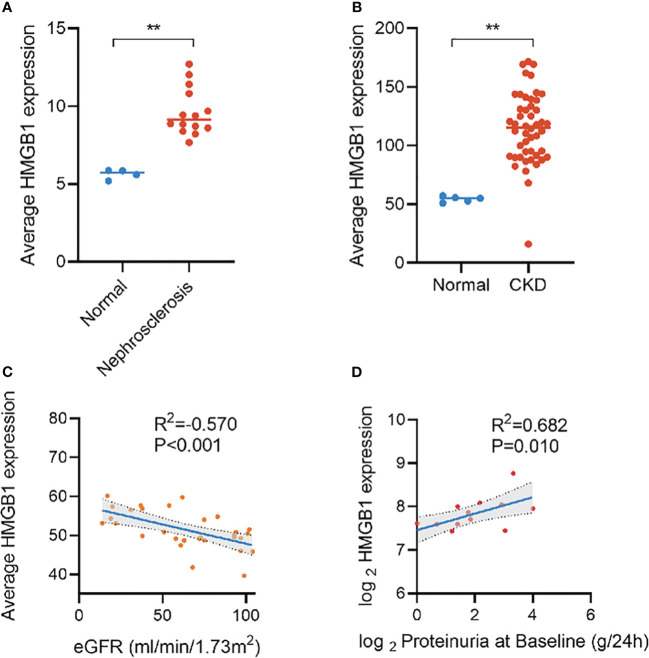
Expression of HMGB1 in different kidney diseases and its correlation with eGFR and proteinuria based on Nephroseq database. **(A)** expression of HMGB1 in nephrosclerosis, **(B)** expression of HMGB1 in CKD, **(C)** correlation between average HMGB1 expression and eGFR, **(D)** correlation between average HMGB1 expression and proteinuria. **P < 0.01, n.s. = no significance.

Over the past decade, many new biomarkers have been evaluated and provided insight into the pathophysiology of kidney disease progression in the context of diabetes. Inflammation is of particular importance (12, 13). Inflammation plays a crucial role in the development and progression of DKD. Persistent inflammation leads to glomerulosclerosis and renal fibrosis, which in turn lead to proteinuria and decreased glomerular filtration rate (14). Numerous inflammation-related molecules, as biomarkers, have been found to be strongly correlated with the progression and prognosis of DKD (13). For example, kidney risk inflammatory signature, composed of 17 proteins including the tumor necrosis factor, is associated with a 10-year risk of ESRD by promoting the inflammatory process of diabetes (15). A study of 2553 participants with normoalbuminuria at a median follow-up of 6.1 years found that TNFR-1 (hazard ratio [HR]=4.2) and TNFR-2 (HR=2.3) were associated with renal outcomes in patients with type 2 diabetes and normoalbuminuria, whereas KIM-1 did not find an association with renal outcomes (16). Similarly, another study reported that TNFR-1 is associated with the risk of kidney failure with replacement therapy in adults with diabetes (HR=1.91) (12). High mobility group box 1 (HMGB1), as an important pro-inflammatory factor, has been found to be elevated in many metabolic and immune diseases, including sepsis (17), rheumatoid arthritis (18), and Alzheimer’s disease (19), and significantly correlated with their progression and prognosis. We recently noticed that HMGB1 activation in kidney disease promotes multiple key events in CKD progression by activating downstream signals, including kidney inflammation, development of persistent fibrosis, kidney aging, acute kidney injury to CKD transition, and important cardiovascular complications (20). Several clinical studies have also shown that elevation of the HMGB1 level is significantly correlated with kidney disease progression (21–23). Furthermore, on the basis of the Nephroseq database, we found an increase of HMGB1 expression in CKD and a significant correlation with eGFR and proteinuria (**Figure 1**). However, the potential role of HMGB1 as a biomarker for the occurrence and progression of DKD has not yet been investigated.

Therefore, in this study, we attempted to investigate the relationship between serum and urine HMGB1 levels and the occurrence and progression of DKD. To verify this, serum tumor necrosis factor receptor superfamily member 1A (TNFR-1) and urine kidney injury molecule (KIM-1), previously reported biomarkers significantly associated with the prognosis of DKD, were also evaluated for comparison.

## 2 Methods

### 2.1 Study design and population

In this cross-sectional study, we recruited 256 participants as the study set from Guang’anmen Hospital of the Chinese Academy of Traditional Chinese Medicine and China-Japan Friendship Hospital from May 2016 to December 2022, including 87 type 2 diabetes mellitus (T2DM) patients without CKD, 109 with DKD, and 60 healthy control participants, and another 42 participants from Dongzhimen Hospital Affiliated to Beijing University of Chinese Medicine (including 15 T2DM patients without CKD, 22 with DKD, and 5 healthy control participants) were included in this study as the validation set. We performed power analysis based on G*Power (Version 3.1.9.7) software to ensure the sample size of each subgroup is sufficient. The inclusion and exclusion criteria of the validation set were the same as those of the study set. The diagnosis of T2DM is based on the guidelines of the American Diabetes Association (ADA) (glycosylated hemoglobin level of 6.5%, fasting blood glucose level of 126 mg/dl, and/or random blood glucose level of 200 mg/dl) (24). The diagnostic criteria of DKD were based on the guidelines of the National Kidney Foundation’s Quality Initiative for Renal Disease Outcomes (NKF-KDOQI) (25), i.e., urinary albumin excretion rate of ≥30 mg/24 hours or abnormal eGFR of <60 mL/min/1.73 m^2^. Individuals with type 1 diabetes, autoimmunity, infection, liver dysfunction, cancer, kidney replacement therapy, or cardiovascular diseases with serious complications were excluded. We also excluded patients with non-DKD, according to previously described (26). Including: 1) diabetes duration less than 5 years; 2) rapid decline of eGFR; 3) rapid increase of urinary albumin or nephrotic syndrome; 4) active urinary sediment (red blood cell, white blood cell or cell tube type); 5) intractable hypertension; 6) combined with other systemic diseases. This study was approved by the Ethics Committee of Guang’anmen Hospital of the Chinese Academy of Traditional Chinese Medicine, Dongzhimen Hospital Affiliated to Beijing University of Chinese Medicine and China-Japan Friendship Hospital respectively, and all participants gave informed consent.

### 2.2 Data collection

All participants were instructed to complete a questionnaire that included their demographic characteristics (age, sex, height, and weight), living habits (smoking and drinking), medical history (duration of type 2 diabetes, hypertension, cardiovascular disease, hyperlipidemia, and fatty liver disease), and standard laboratory functional test results (values of hemoglobin, urinary microalbumin, and 24-hour urine protein quantification). eGFR was evaluated according to the 2009 Chronic Kidney Disease Epidemiology Collaboration formula (27). Blood and urine samples were obtained from all participants. Blood samples were centrifuged at 3000 rpm for 10 minutes, and the supernatant was kept at -80 °C. Urine samples were centrifuged at 1500 rpm for 10 minutes, and the supernatant was kept at -80 °C.

### 2.3 Laboratory tests of HMGB1

Both serum and urine HMGB1 levels were detected using the human high-mobility group protein B1 (HMGB1) enzyme-linked immunosorbent assay (ELISA) kit (E-EL-H1554c, Elabscience), and ELISA was performed according to the manufacturer’s instructions. The coefficient of inter-assay and intra-assay variation was <10%. All measurements were repeated thrice horizontally, and the average of these values was used to prevent measurement bias. Additionally, urine HMGB1 levels were corrected for urinary creatinine levels.

### 2.4 Measurement of TNFR-1 and KIM-1 levels

According to the manufacturer’s instructions, the human tumor necrosis factor receptor superfamily member 1A (TNFRSF1A) ELISA kit (E-EL-H0217c, Elabscience) and the human kidney injury molecule 1 (KIM-1) ELISA kit (E-EL-H6029, Elabscience) were used to quantify the contents of TNFR-1 in the serum samples and KIM-1 in the urine samples, respectively. The coefficient of inter-assay and intra-assay variation was <10%. All samples were measured thrice horizontally, and the average of these values was used to prevent measurement bias. Moreover, urinary KIM-1 levels were corrected for urinary creatinine levels.

### 2.5 Statistical analysis

Descriptive statistics were used to determine the baseline characteristics of the study population. The results are reported using descriptive statistical methods, where the continuous variables are continuously presented as variables with normal or non-normal distribution through means and standard deviations (SD) or medians and interquartile ranges (IQRs), and categorical variables are presented as quantities and percentages. We used t-tests, one-way analysis of variance, and Kruskal–Wallis tests to analyze the relationship between continuous variables for comparison between different groups; the chi-square test was used to analyze the relationship between categorical variables. Correlations between different indicators were tested using the Spearman correlation test. The relationship between serum HMGB1 and CKD stage was tested using ordinal logistic regression. While, some potential confounders were corrected in the multivariate regression models. Receiver-operator characteristic (ROC) curves were drawn, and the performance of serum and urine HMGB1 were evaluated by the area under the curve (AUC). Considering the possible non-monotonic effects between serum HMGB1 and renal function progression in DKD, we also used restricted cubic spline (RCS) fitting univariate and multivariate regression models to flexibly model and visualize the nonlinear relationship between serum HMGB1 levels and the risk of DKD progression (28). To get the best fitting effect, we fitted the model with the number of knots between 3-7 separately, and selected the knots corresponding to the lowest value for the akaike information criterion (AIC) as the number of knots. We set the median of the first quartile of serum HMGB1 as the reference. To test for potential nonlinearity, a likelihood ratio test was used to compare the RCS model with a model containing only linear terms. A two-tailed p-value of <0.05 was considered significant. All statistical analyses were performed using SPSS statistics 25.0 (SPSS, Inc., Chicago, IL, USA) and R software, version 4.2.2, along with MSTATA software (www.mstata.com).

## 3 Results

### 3.1 Basic characteristics of the study population

The demographic characteristics of the participants are presented in **Table 1**. Among the 256 participants, 109 had DKD, 87 had T2DM, and 60 were healthy control, and there were 130 males (50.78%) with an average age of 57.71 ± 9.69 years and a body mass index (BMI) of 24.70 ± 3.36 kg/m^2^. Less than a quarter of the participants reported smoking (20.31%) or drinking (21.48%).

**Table 1:** Baseline demographic and clinical characteristics of the study population.

Characteristics	Total (n=256)	Healthy control (n=60)	T2DM (n=87)	DKD (n=109)	*p*-value
Age (years)	57.71 ± 9.69	55.43 ± 10.00	55.51 ± 10.18	60.72 ± 8.28	<0.001
Male (%)	130 (50.78)	22 (36.67)	41 (47.13)	67 (61.47)	0.006
BMI (kg/m^2^)	24.70 ± 3.36	22.99 ± 2.71	24.99 ± 3.55	25.42 ± 3.24	<0.001
Duration of diabetes (years)	12.30 ± 8.06	/	9.31 ± 8.43	13.77 ± 7.61	<0.001
Hypertension (%)	143 (55.86)	3 (5.00)	46 (52.87)	94 (86.24)	<0.001
CVD (%)	86 (33.59)	9 (15.00)	31 (35.63)	46 (42.20)	0.001
Hyperlipidemia (%)	122 (46.48)	6 (10.00)	50 (57.47)	66 (60.55)	<0.001
Smokers (%)	52 (20.31)	3 (5.00)	19 (21.84)	30 (27.52)	0.002
Drinkers (%)	55 (21.48)	5 (8.33)	24 (27.59)	26 (23.85)	0.015
HGB (g/L)	132.09 ± 20.44	139.28 ± 14.23	135.79 ± 18.87	125.17 ± 22.46	<0.001
HbA1c (%)	7.47 ± 1.91	6.18 ± 1.18	7.96 ± 1.97	7.78 ± 1.87	<0.001
ALT (U/L)	18.00 (14.00,27.00)	18.00 (13.00,29.00)	20.00 (15.00,28.70)	17.80 (13.40,24.00)	0.104
AST (U/L)	19.00 (15.93,23.45)	20.00 (17.00,25.00)	18.90 (15.20,24.00)	18.20 (14.00,23.00)	0.116
ALB (g/L)	41.26 ± 5.71	44.37 ± 3.22	43.38 ± 2.98	37.84 ± 6.59	<0.001
Scr (μmol/L)	98.57 ± 87.03	58.55 ± 11.15	63.59 ± 17.04	148.53 ± 114.85	<0.001
eGFR (ml/min/1.73m^2^)	82.93 ± 30.66	102.06 ± 9.06	98.35 ± 14.09	60.10 ± 33.10	<0.001
Urea (mmol/L)	8.07 ± 6.56	4.95 ± 1.17	5.91 ± 2.26	11.50 ± 8.70	<0.001
UA (μmol/L)	344.98 ± 87.87	307.46 ± 70.92	343.53 ± 88.17	366.79 ± 89.60	<0.001
TC (mmol/L)	4.95 ± 1.49	4.91 ± 1.33	4.77 ± 1.45	5.11 ± 1.59	0.594
TG (mmol/L)	1.78 ± 1.01	1.34 ± 0.65	1.88 ± 1.17	1.94 ± 0.97	<0.001
LDL-C (mmol/L)	2.97 ± 1.02	2.90 ± 0.93	3.01 ± 1.03	2.97 ± 1.07	0.742
HDL-C (mmol/L)	1.28 ± 0.42	1.37 ± 0.33	1.22 ± 0.31	1.28 ± 0.52	0.011
hs-CRP (mg/L)	1.32 (0.65,2.30)	1.02 (0.82,2.60)	1.36 (0.66,2.23)	1.37 (0.50,2.15)	0.937
UACR (mg/g)	20.00 (4.08,355.48)	5.74 (2.96,15.04)	3.73 (0.80,9.21)	539.70 (77.44,959.61)	<0.001
24h-UTP (mg/24h)	110.24 (60.05,1660.00)	49.31 (25.35,82.28)	82.09 (60.00,110.00)	2250.00 (475.00,4645.00)	<0.001
Serum HMGB1 (ng/ml)	33.55 ± 25.99	19.81 ± 10.01	19.87 ± 12.82	52.03 ± 28.45	<0.001
Urine HMGB1/Cr (pg/ug)	3.77 (2.19,7.93)	2.30 (1.25,4.91)	2.96 (2.01,4.56)	7.72 (3.8,12.13)	<0.001
KIM-1/Cr (pg/ug)	0.45 (0.24,0.90)	0.32 (0.16,0.48)	0.30 (0.18,0.49)	0.94 (0.51,1.66)	<0.001
TNFR-1 (ng/ml)	2.45 ± 0.76	2.10 ± 0.41	2.21 ± 0.34	2.84 ± 0.95	<0.001

BMI, body-mass index; HGB, hemoglobin; HbA1c, glycated haemoglobin A1c; ALT, alanine transaminase; AST, aspartate aminotransferase; ALB, albumin; Scr, serum creatinine; eGFR, estimated glomerular filtration rate; UA, uric acid; TC, total cholesterol; TG, triglycerides; HDL-C, high density lipoprotein cholesterol; LDL-C, low density lipoprotein cholesterol; hs-CRP, high-sensitivity C-reactive protein; UACR, Urinary albumin/creatinine ratio; UTP, urinary total protein; HMGB1, high mobility group box protein 1; TNFR-1, tumor necrosis factor receptor superfamily member 1A; KIM-1, kidney injury molecule-1; CVD, cardiovascular diseases; DKD, diabetic kidney disease.

Compared with T2DM patients without CKD and healthy controls, patients with DKD were older and there was significant predominance in male sex, duration of diabetes, hypertension, cardiovascular disease, smoking, and alcohol consumption. Among the population with DKD, while there was no statistically significant difference in LDL-C and hs-CRP between T2DM patients without CKD and healthy controls.

### 3.2 Elevation of the HMGB1 level in the population with DKD

**Figure 2:**
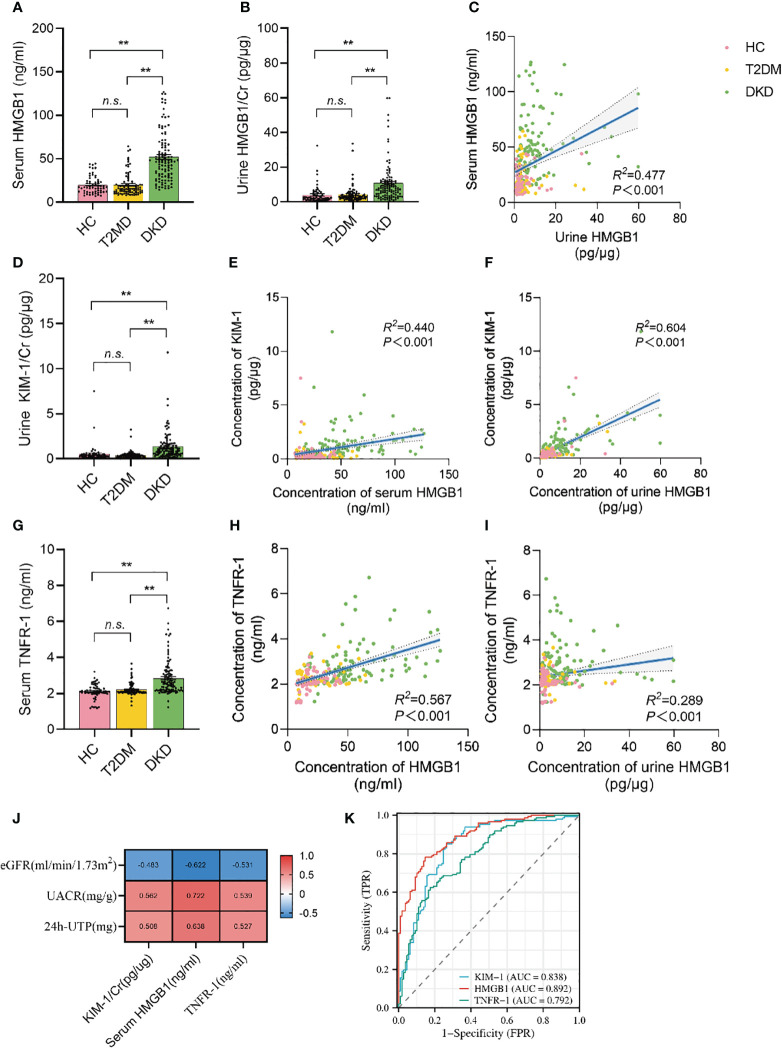
Serum and urine HMGB1 were significantly elevated in DKD population and correlated with TNFR-1 and KIM-1. **(A)** serum HMGB1 levels in the study population, **(B)** urine HMGB1 levels in the study population, **(C)** correlation between serum and urine HMGB1, **(D)** urine KIM-1/Cr levels in the study population, **(E)** correlation between serum HMGB1 and urine KIM-1/Cr, **(F)** correlation between urine HMGB1 and KIM-1/Cr, **(G)** serum TNFR-1 levels in the study population, **(H)** correlation between serum HMGB1 and TNFR-1, **(I)** correlation between urine HMGB1 and serumTNFR-1, **(J)** correlation between serum HMGB1, urine KIM-1 and serum TNFR-1 and proteinuria and renal function progression, **(K)** the performance of serum HMGB1, urine KIM-1 and serum TNFR-1 to identify DKD based on the ROC curve. n.s. = no significance.

The serum HMGB1 levels in the population with DKD was significantly higher than that in the population with long-term diabetes but without CKD or in healthy control people without diabetes (52.03 ± 28.45 *v.s.* 19.87 ± 12.82 and 19.81 ± 10.01) (**Figure 2A**). Urine HMGB1 levels corrected by urinary creatinine levels showed results similar to serum HMGB1 levels (**Figure 2B**). Notably, we found a significant positive correlation between serum and urine HMGB1 levels in this population (*R* ^2^ = 0.477, *p*<0.001) (**Figure 2C**).

Urine KIM-1 is a known and confirmed marker of renal tubular injury. Consistent with previous reports (29), we found that KIM-1 levels corrected for urinary creatinine levels were significantly elevated in the population with DKD (**Figure 2D**), and there was a significant correlation between serum HMGB1 and urine KIM-1 levels (*R* ^2^ = 0.440, *p*<0.001) (**Figure 2E**). Similarly, creatinine-corrected urinary HMGB1 levels were significantly correlated with KIM-1 levels (*R* ^2^ = 0.604, *p*<0.001) (**Figure 2F**).

More importantly, recent study reported that TNFR-1 expression is strongly correlated with the prognosis of DKD. Every unit of TNFR-1 increased the risk of DKD renal function decline by 117% (30). We also found that serum TNFR-1 levels were significantly elevated in the population with DKD (2.84 ± 0.95 *v.s.* 2.21 ± 0.34 and 2.10 ± 0.41) (**Figure 2G**), and there was a significant correlation between serum HMGB1 and serum TNFR-1 levels (*R* ^2^ = 0.567, *p*<0.001) (**Figure 2H**). Moreover, there was a significant correlation between creatinine-corrected urine HMGB1 levels and serum TNFR-1 levels (**Figure 2I**). (*R* ^2^ = 0.289, *p*<0.001). More importantly, we found that serum HMGB1 showed a higher correlation with proteinuria and renal function progression compared to urine KIM-1 and serum TNFR-1(**Figure 2J**), and its efficacy in predicting DKD occurrence was better (**Figure 2K**).

### 3.3 Association of the HMGB1 levels with DKD‐related traits

**Figure 3:**
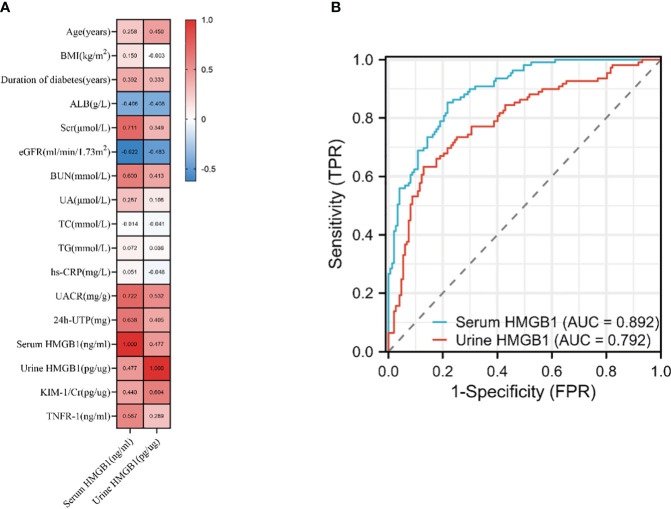
Association of the HMGB1 levels with DKD‐related traits. **(A)** correlation of serum and urine HMGB1 and DKD related variables based on Spearman correlation test, **(B)** the performance of serum and urine HMGB1 to identify DKD based on the ROC curve.

Next, we found that the levels of HMGB1 in serum and urine are related to age, duration of diabetes, the proportion of hypertension and CVD (**Table 2**, **Figure 2A**). In addition, serum HMGB1 is also related to sex and smoking status (**Table 2**). We further found that serum and urine HMGB1 levels were significantly correlated with clinical indicators related to DKD (**Table 2**, **Figure 3A**), and the diagnostic efficacy of serum HMGB1 for the occurrence of DKD was better than that of corrected urine HMGB1 (AUC=0.892 *v.s.* AUC=0.792) (**Figure 3B**). Therefore, serum HMGB1 levels were selected for further analyses.

**Table 2:** Association of serum HMGB1 with various patient characteristics.

Variables		Serum HMGB1		Urine HMGB1	
		Mean± SD or R correlation factor	*p*-value	Mean± SD or R correlation factor	*p-*value
Age (years)		0.258	<0.001	0.450	<0.001
Sex	Male (n=130)	42.39 ± 28.30	<0.001	3.82 (2.07,7.98)	0.914
	Female (n=126)	24.42 ± 19.66		3.67 (2.14,7.72)	
BMI (kg/m^2^)		0.150	0.016	-0.003	0.965
Duration of diabetes (years)		0.392	<0.001	0.333	<0.001
Hypertension	Yes (n=143)	43.86 ± 29.44	<0.001	5.62 (3.03,10.13)	<0.001
	No (n=113)	20.49 ± 11.48		2.56 (1.66,5.31)	
CVD	Yes (n=86)	39.02 ± 28.50	0.023	5.52 (3.03,8.97)	0.003
	No (n=170)	30.77 ± 24.25		3.23 (1.96,6.87)	
Hyperlipidemia	Yes (n=122)	35.06 ± 26.51	0.375	4.51 (2.37,8.91)	0.109
	No (n=134)	32.17 ± 25.54		3.37 (2.07,6.76)	
Smokers	Yes (n=52)	45.40 ± 28.63	<0.001	3.82 (2.20,8.91)	0.533
	No (n=204)	30.52 ± 24.45		3.68 (2.14,7.56)	
Drinkers	Yes (n=55)	36.55 ± 21.69	0.335	3.50 (2.15,6.49)	0.293
	No (n=201)	32.72 ± 27.04		4.09 (2.19,8.08)	
ALB (g/L)		-0.406	<0.001	-0.408	<0.001
Scr (μmol/L)		0.711	<0.001	0.349	<0.001
eGFR (ml/min/1.73m^2^)		-0.622	<0.001	-0.483	<0.001
Urea (mmol/L)		0.600	<0.001	0.413	<0.001
UA (μmol/L)		0.287	<0.001	0.106	0.091
TC (mmol/L)		-0.014	0.819	-0.041	0.516
TG (mmol/L)		0.072	0.252	0.036	0.568
hs-CRP (mg/L)		0.051	0.412	-0.048	0.448
UACR (mg/g)		0.722	<0.001	0.532	<0.001
24h-UTP (mg)		0.638	<0.001	0.405	<0.001
CKD stage	G1 (n=153) (eGFR≥ 90)	21.18 ± 11.92	<0.001	2.83 (1.70,5.37)	<0.001
	G2 (n=50) (60≤eGFR < 90)	32.24 ± 18.97		4.59 (2.68,6.78)	
	G3 (n=27) (30≤eGFR < 60)	62.46 ± 21.96		9.96 (5.26,12.81)	
	G4 (n=17) (15≤eGFR < 30)	73.08 ± 28.40		8.97 (7.72,16.68)	
	G5 (n=9) (eGFR <15)	89.61 ± 28.92		9.53 (7.98,22.53)	
Serum HMGB1 (ng/ml)		1	/	0.477	<0.001
Urine HMGB1 (pg/ug)		0.477	<0.001	1	/
KIM-1/Cr (pg/ug)		0.440	<0.001	0.604	<0.001
TNFR-1 (ng/ml)		0.567	<0.001	0.289	<0.001

BMI, body-mass index; ALB, albumin; Scr, serum creatinine; eGFR, estimated glomerular filtration rate; UA, uric acid; TC, total cholesterol; TG, triglycerides; HDL-C, high density lipoprotein cholesterol; LDL-C, low density lipoprotein cholesterol; hs-CRP, high-sensitivity C-reactive protein; UACR, urinary albumin/creatinine ratio; UTP, urinary total protein; HMGB1, high mobility group box protein 1; TNFR-1, tumor necrosis factor receptor superfamily member 1A; KIM-1, kidney injury molecule-1; CVD, cardiovascular diseases; CKD, chronic kidney disease.

### 3.4 Correlations between serum HMGB1 and other variables

To determine the relationship between serum HMGB1 levels and DKD progression-related variables, we divided the included population into four groups according to the quartiles of serum HMGB1 levels, and the baseline characteristics of the included participants are shown in **Table 3**. Age, male proportion, duration of diabetes, hypertension proportion, CVD proportion, smokers’ proportion, and serum TNFR-1 levels increased with increasing serum HMGB1 levels (all *p* values < 0.05). Additionally, HGB levels decreased in the highest quartile of serum HMGB1 levels. The differences in blood lipid levels were not statistically significant (all *p* values > 0.05). Importantly, we found that an increase in the quartile of serum HMGB1 levels was associated with an increased incidence of renal function decline and an increased number of patients with higher CKD stages compared with the lowest quartile (**Table 3**) (all *p* values < 0.05). This indicates that there is a dose-response relationship between serum HMGB1 levels and the renal progression of DKD.

**Table 3:** Clinical and biochemical parameters for participants, according to quartile of Serum HMGB1 levels.

Characteristics	Total	Q1 (<15.20)	Q2 (15.20-23.62)	Q3 (23.62-46.12)	Q4 (>46.12)	*p-*value
N	256	64	64	64	64	/
Age (years)	57.71 ± 9.69	54.86 ± 9.41	57.34 ± 9.33	57.84 ± 10.23	60.80 ± 9.05	0.006
Male (%)	130 (50.78)	13 (20.31)	28 (43.75)	44 (68.75)	45 (70.31)	<0.001
BMI (kg/m^2^)	24.70 ± 3.36	24.45 ± 3.12	23.90 ± 3.45	24.92 ± 3.64	25.53 ± 3.08	0.042
Duration of diabetes (years)	11.78 ± 8.27	7.94 ± 7.62	10.90 ± 9.03	11.15 ± 7.73	15.32 ± 7.25	<0.001
Hypertension (%)	143 (55.86)	20 (31.25)	27 (42.19)	36 (56.25)	60 (93.75)	<0.001
CVD (%)	86 (33.59)	17 (26.56)	17 (26.56)	22 (34.38)	30 (46.88)	0.015
Hyperlipidemia (%)	122 (47.66)	33 (51.56)	24 (37.50)	28 (43.75)	37 (57.81)	0.134
Smokers (%)	52 (20.31)	4 (6.25)	12 (18.75)	13 (20.31)	23 (35.94)	0.001
Drinkers (%)	55 (21.48)	9 (14.06)	11 (17.19)	19 (29.69)	16 (25.00)	0.121
HGB (g/L)	132.09 ± 20.44	132.98 ± 18.46	137.02 ± 13.92	140.19 ± 18.02	118.17 ± 23.30	<0.001
HbA1c (%)	7.47 ± 1.91	7.49 ± 1.92	7.46 ± 2.26	7.53 ± 2.00	7.39 ± 1.41	0.978
ALT (U/L)	18.00 (14.00,27.00)	17.00 (14.00,27.00)	18.00 (16.00,25.60)	21.00 (14.10,31.00)	15.50 (13.00,22.80)	0.05
AST (U/L)	19.00 (15.93,23.45)	18.00 (15.00,22.00)	19.00 (16.00,24.00)	19.30 (16.00,25.00)	18.20 (15.00,22.00)	0.304
ALB (g/L)	41.26 ± 5.71	43.32 ± 2.85	43.28 ± 3.94	42.37 ± 4.91	36.06 ± 6.86	<0.001
Scr (μmol/L)	98.57 ± 87.03	56.38 ± 11.67	63.10 ± 14.89	82.35 ± 39.19	192.46 ± 128.28	<0.001
eGFR (ml/min/1.73m^2^)	82.93 ± 30.66	101.95 ± 11.54	97.37 ± 11.97	86.49 ± 24.78	45.92 ± 30.50	<0.001
Urea (mmol/L)	8.07 ± 6.56	5.17 ± 1.87	5.63 ± 1.43	7.21 ± 4.39	14.26 ± 9.76	<0.001
UA (μmol/L)	344.98 ± 87.87	320.61 ± 77.96	334.31 ± 89.53	349.97 ± 102.59	375.02 ± 70.53	0.003
TC (mmol/L)	4.95 ± 1.49	4.93 ± 1.52	4.91 ± 1.32	4.72 ± 1.32	5.22 ± 1.75	0.314
TG (mmol/L)	1.78 ± 1.01	1.92 ± 1.25	1.54 ± 0.84	1.79 ± 1.05	1.86 ± 0.78	0.157
LDL-C (mmol/L)	2.97 ± 1.02	3.16 ± 1.09	2.93 ± 0.92	2.79 ± 0.91	2.98 ± 1.13	0.225
HDL-C (mmol/L)	1.28 ± 0.42	1.24 ± 0.27	1.38 ± 0.37	1.25 ± 0.37	1.27 ± 0.58	0.211
hs-CRP (mg/L)	1.32 (0.65,2.30)	1.31 (0.68,2.21)	1.04 (0.59,1.86)	1.42 (0.79,2.54)	1.59 (0.50,2.32)	0.352
UACR (mg/g)	20.00 (4.08,355.48)	3.33 (0.69,9.40)	13.22 (3.95,24.53)	51.48 (5.84,207.18)	734.85 (358.72,1107.01)	<0.001
24h-UTP (mg)	110.24 (60.05,1660.00)	72.81 (60.05,95.26)	87.55 (50.00,143.48)	195.00 (43.53,1082.5)	3680.00 (2255.00,5445.00)	<0.001
CKD stage						
G1 (%) (eGFR≥90)	153 (59.77)	55 (85.94)	51 (79.69)	39 (60.94)	8 (12.50)	<0.001
G2 (%) (60≤eGFR<90)	50 (19.53)	9 (14.06)	13 (20.31)	17 (26.56)	11 (17.19)	0.323
G3 (%) (30≤eGFR <60)	27 (10.55)	0 (0.00)	0 (0.00)	6 (9.38)	21 (32.81)	<0.001
G4 (%) (15≤eGFR<30)	17 (6.64)	0 (0.00)	0 (0.00)	2 (3.12)	15 (23.44)	<0.001
G5 (%) (eGFR<15)	9 (3.51)	0 (0.00)	0 (0.00)	0 (0.00)	9 (14.06)	<0.001
Serum HMGB1 (ng/ml)	33.55 ± 25.99	11.19 ± 2.15	18.59 ± 2.35	34.13 ± 6.79	70.27 ± 23.95	<0.001
Urine HMGB1 (pg/ug)	3.77 (2.19,7.93)	2.42 (1.30,4.05)	2.89 (2.07,6.04)	4.92 (2.25,9.63)	7.96 (4.82,12.70)	<0.001
KIM-1/Cr (pg/ug)	0.45 (0.24,0.90)	0.33 (0.22,0.54)	0.33 (0.18,0.50)	0.41 (0.19,1.02)	1.10 (0.66,1.72)	<0.001
TNFR-1 (ng/ml)	2.45 ± 0.76	2.05 ± 0.36	2.25 ± 0.40	2.40 ± 0.52	3.12 ± 1.04	<0.001

BMI, body-mass index; HGB, hemoglobin; HbA1c, glycated haemoglobin A1c; ALT, alanine transaminase; AST, aspartate aminotransferase; ALB, albumin; Scr, serum creatinine; eGFR, estimated glomerular filtration rate; UA, uric acid; TC, total cholesterol; TG, triglycerides; HDL-C, high density lipoprotein cholesterol; LDL-C, low density lipoprotein cholesterol; hs-CRP, high-sensitivity C-reactive protein; UACR, urinary albumin/creatinine ratio; UTP, urinary total protein; HMGB1, high mobility group box protein 1; TNFR-1, tumor necrosis factor receptor superfamily member 1A; KIM-1, kidney injury molecule-1; CVD, cardiovascular diseases; CKD, chronic kidney disease.

### 3.5 Dose-response relationships of serum HMGB1 with the risk of kidney function decline in DKD

**Figure 4:**
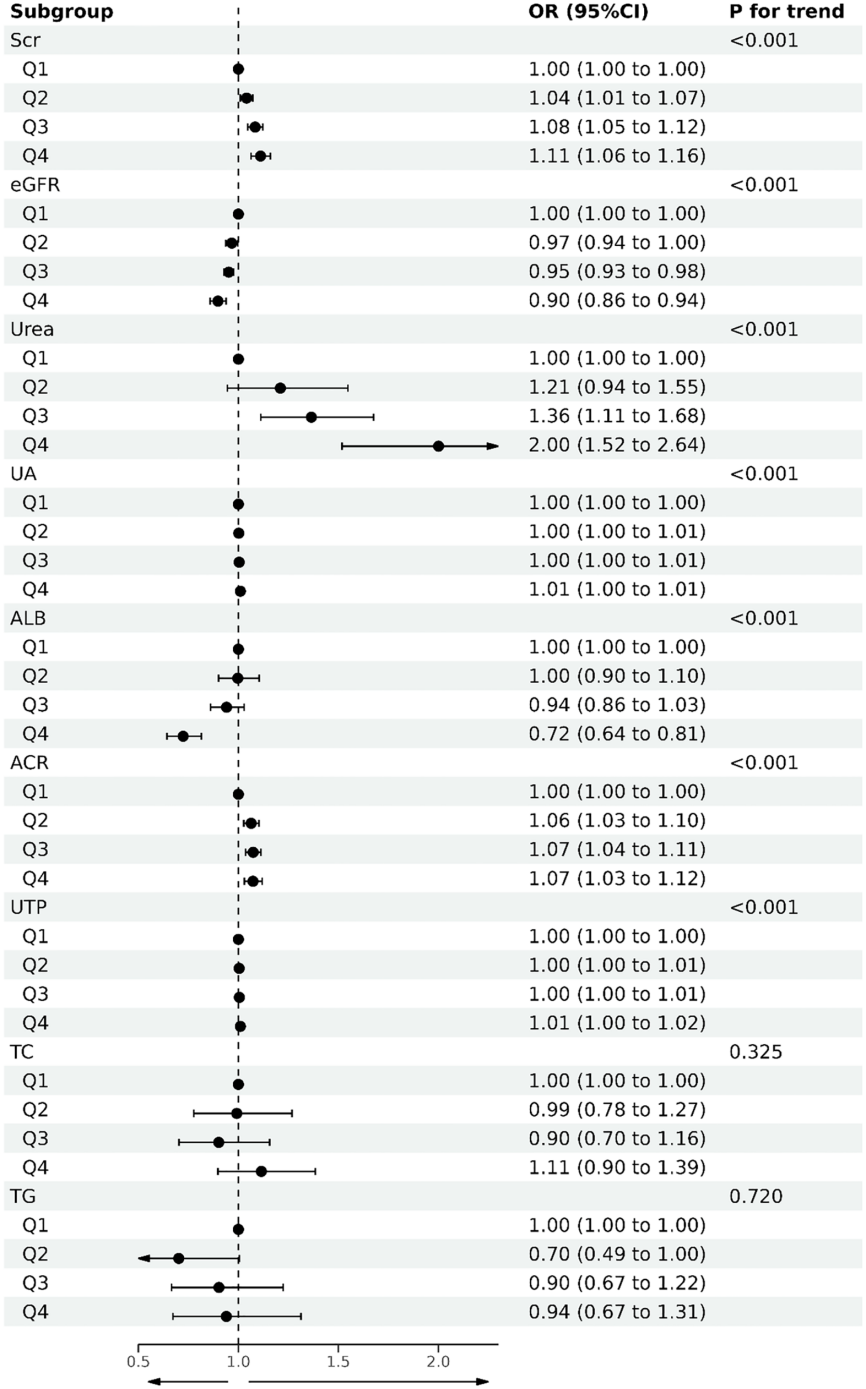
Forest plot of quartile of serum HMGB1 levels and DKD-related variables based on logistic regression.

Based on univariate and multivariate logistic regression, we found that higher HMGB1 levels significantly increased the risk of renal function decline compared with the first quartile (**Table 4**, **Figure 4**), and we constructed three additional multivariate logistic regression models to adjust for confounding variables. As **Table 4** shows, elevated serum HMGB1 levels remained an independent risk factor for renal function decline in DKD.

**Table 4:** OR and 95% CI for kidney function decline in each quartile based on univariate and multivariate logistic regression.

Serum HMGB1, ng/ml	Crude		Model 1		Model 2		Model 3	
	OR (95% CI)	*p-*value	OR (95% CI)	*p-*value	OR (95% CI)	*p-*value	OR (95% CI)	*p-*value
Serum HMGB1	1.071(1.058,1.085)	<0.001	1.070(1.054,1.086)	<0.001	1.067(1.050,1.083)	<0.001	1.043(1.024,1.061)	<0.001
Q1-Q2(<23.62)	1.080(0.970,1.204)	0.161	1.127(0.958,1325)	0.150	1.059(0.886,1.265)	0.532	1.030(0.848,1.250)	0.769
Q3-Q4(>23.62)	1.062(1.045,1.080)	<0.001	1.066(1.046,1.087)	<0.001	1.066(1.046,1.088)	<0.001	1.047(1.024,1.069)	<0.001
*p* for trend	<0.001		<0.001		<0.001		<0.001	

Model 1: adjusted for age, sex, BMI and duration of diabetes.

Model 2: further adjusted for hypertension, hyperlipidemia, CVD and smokers.

Model 3: further adjusted for ALB, UA, KIM-1/Cr, TNFR-1.

BMI, body-mass index; ALB, albumin; UA, uric acid; HMGB1, high mobility group box protein 1; TNFR-1, tumor necrosis factor receptor superfamily member 1A; KIM-1, kidney injury molecule-1; CVD, cardiovascular diseases; DKD, diabetic kidney disease; OR, odds ratio; CI, confidence interval.

**Figure 5:**
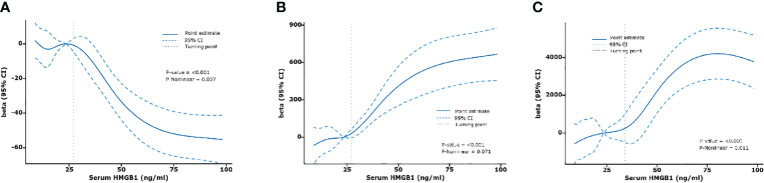
Relationships between serum HMGB1 and renal function fitted with restricted cubic spline (RCS) models. The solid lines indicate multivariate-adjusted Betas, and dotted lines indicate the 95% CIs derived from restricted cubic spline regression. Adjusted for age, sex, hypertension, smokers, and duration of diabetes. **(A)** serum HMGB1 and eGFR, **(B)** serum HMGB1 and UACR, **(C)** serum HMGB1 and UTP.

Next, we constructed an RCS model to flexibly visualize the relationship between serum HMGB1 levels and DKD progression on a continuous scale with or without correction for covariates. We observed an inverted J-shaped nonlinear relationship between serum HMGB1 levels and eGFR (*p*-nonlinear=0.007, *p*<0.001, knots=6, AIC=2137.97), after excluding the confounding factors of age, sex, hypertension, smokers, and duration of diabetes. When the concentration of HMGB1 was <27 ng/ml, the risk of renal function decline in DKD was stable (OR per SD=0.82, *p*=0.017). In contrast, when the concentration of serum HMGB1 was ≥27 ng/ml, the risk of renal function decline rapidly increases (OR per SD=0.53, *p*<0.001) (**Table 5**, **Figure 5A**). When adjusting for confounders, we found no nonlinear relationship between serum HMGB1 and UACR (*p*-nonlinear=0.071, *p*<0.001, knots=5, AIC=3500.54, **Table 5**, **Figure 5B**. Additionally, a similar relationship was observed between serum HMGB1 and UTP (*p*-nonlinear=0.011, *p*<0.001, knots=7, AIC=4417.18). Serum HMGB1 and UTP also showed a J-shaped relationship, after excluding the confounding factors of age, sex, hypertension, smokers, and duration of diabetes (**Table 5**, **Figure 5C**).

**Table 5:** Effect of standardized serum HMGB1 level on eGFR adjusted coefficients from segmented linear regression analysis.

Characteristic	Serum HMGB1, ng/ml	OR per SD	95% CI	*p*-value
eGFR	< 27	0.82	0.69,0.96	0.017
	≥ 27	0.53	0.46,0.62	<0.001
UACR	< 27	1.51	1.30,1.75	<0.001
	≥ 27	1.62	1.38,1.90	<0.001
UTP	< 27	1.36	1.17,1.57	<0.001
	≥ 27	1.49	1.23,1.80	<0.001

eGFR, estimated glomerular filtration rate; UACR, urinary albumin/creatinine ratio; UTP, urinary total protein; HMGB1, high mobility group box protein 1; OR, odds ratio; CI, confidence interval.

OR were adjusted for sex, hypertension, smokers, duration of diabetes, and age.

### 3.6 Subgroup and sensitivity analyses

Subgroup analysis showed that the association between HMGB1 and the risk of DKD progression was not significantly affected in the data stratified by age, sex, and BMI. The sensitivity analysis did not reveal substantial changes in the results after excluding participants with CVD, hypertension, and smokers, respectively, indicating that our results are robust.

### 3.7 Validation set

**Figure 6:**
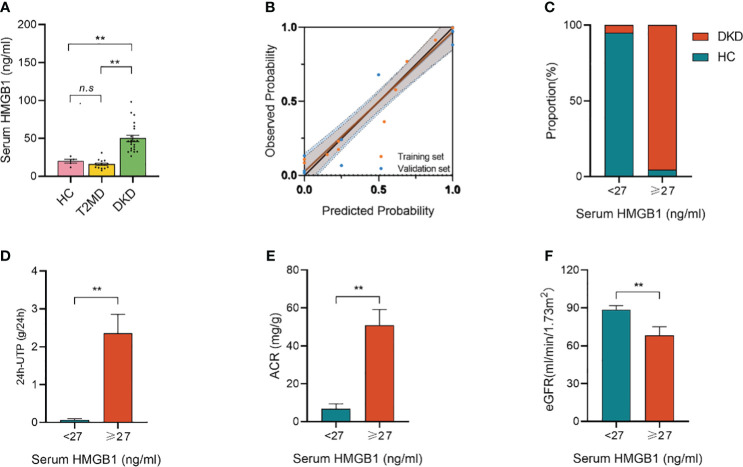
Validation set from another independent center. **(A)** serum HMGB1 levels in different groups; **(B)** Calibration plot comparing the observed and predicted probabilities for kidney function decline from the logistic regression for the study and validation set; **(C)** Proportion of DKD population with serum HMGB1 ≥27 ng/ml or <27 ng/ml; **(D)** 24h-UTP levels in serum HMGB1 ≥27 ng/ml or <27 ng/ml; **(E)** ACR levels in serum HMGB1 ≥27 ng/ml or <27 ng/ml; **(F)** eGFR levels in serum HMGB1 ≥27 ng/ml or <27 ng/ml. **P < 0.01, n.s. = no significance.

To validate the role of serum HMGB1, we analyzed the data of 42 participants recruited independently in another center. As shown in **Table 6**, the study set and validation set had similar baseline characteristics. The median levels of baseline serum HMGB1 were similar in both sets. Similar findings were observed in the validation set: serum HMGB1 levels were also higher in patients with DKD compared with patients with long-term diabetes but without CKD nephropathy or in healthy control people without diabetes (**Figure 6A**). The calibration plot showed that there was a good fit between the observed and predicted probabilities, and no statistical difference from the perfect fit was found for the study (*p=*0.202) and the validation set (p=0.825) (**Figure 6B**). At the same time, according to the above threshold, we also grouped the population whether the serum HMGB1 ≥27 ng/ml. We found that the proportion of DKD was significantly increased (**Figure 6C**), the level of 24h-UTP and ACR were significantly increased (**Figures 6D, E**), and the level of GFR was significantly decreased (**Figure 6F**) in the population with serum HMGB1 ≥27 ng/ml.

**Table 6:** Baseline characteristics of participants included in the validation set compared to the study set.

Characteristics	Study set (n=256)	Validation set (n=42)	*p*-value
DKD (%)	109 (42.58)	22 (52.38)	0.235
Age (years)	57.71 ± 9.69	59.05 ± 8.45	0.400
Male (%)	130 (50.78)	25 (59.52)	0.293
BMI (kg/m^2^)	24.70 ± 3.36	24.96 ± 3.96	0.657
Duration of diabetes (years)	12.30 ± 8.06	11.20 ± 8.90	0.454
Hypertension (%)	143 (55.86)	30 (71.42)	0.058
CVD (%)	86 (33.59)	10 (23.81)	0.209
Hyperlipidemia (%)	122 (46.48)	28 (83.33)	0.022
Smokers (%)	52 (20.31)	10 (23.81)	0.605
Drinkers (%)	55 (21.48)	6 (15.00)	0.346
HGB (g/L)	132.09 ± 20.44	133.62 ± 17.80	0.648
HbA1c (%)	7.47 ± 1.91	7.63 ± 1.77	0.627
ALT (U/L)	18.00 (14.00,27.00)	18.00 (13.00,22.00)	0.787
AST (U/L)	19.00 (15.93,23.45)	17.00 (13.60,20.00)	0.076
ALB (g/L)	41.26 ± 5.71	40.37 ± 6.30	0.360
Scr (μmol/L)	98.57 ± 87.03	107.15 ± 89.78	0.556
eGFR (ml/min/1.73m^2^)	82.93 ± 30.66	77.81 ± 26.62	0.319
Urea (mmol/L)	8.07 ± 6.56	8.30 ± 4.42	0.824
UA (μmol/L)	344.98 ± 87.87	364.61 ± 83.74	0.178
TC (mmol/L)	4.95 ± 1.49	4.70 ± 1.05	0.313
TG (mmol/L)	1.78 ± 1.01	1.72 ± 0.82	0.735
LDL-C (mmol/L)	2.97 ± 1.02	2.97 ± 0.97	0.984
HDL-C (mmol/L)	1.28 ± 0.42	1.34 ± 0.40	0.426
UACR (mg/g)	20.00 (4.08,355.48)	25.00 (3.61,428.32)	0.761
24h-UTP (mg/24h)	110.24 (60.05,1660.00)	350.00 (30.00,1720.00)	0.216
Serum HMGB1 (ng/ml)	33.55 ± 25.99	30.72 ± 23.21	0.513
KIM-1/Cr (pg/ug)	0.45 (0.24,0.90)	0.50 (0.28,0.89)	0.509
TNFR-1 (ng/ml)	2.45 ± 0.76	2.26 ± 0.49	0.108

## 4 Discussion

Liquid biopsy is increasingly used for early diagnosis and precision medicine (31), especially for cancer (32). More importantly, advances in omics techniques and computational analysis have increased the sensitivity, specificity, and accuracy of liquid biopsies (33, 34). In kidney disease, the use of this noninvasive fluid biopsy compensates for the risk of bleeding, pain, infection, and renal vein thrombosis associated with invasive kidney biopsy and the limitation of not being able to perform repeat kidney biopsies to monitor disease progression (35). However, for DKD, the number of biomarkers for identifying renal disease progression and poor prognosis remains limited. Therefore, the identification of specific and sensitive biomarkers is essential for early diagnosis and disease management. For example, a urine proteomics-based study found that urinary CKD273, composed of 273 peptides, has great potential for predicting kidney outcomes in diabetes (36, 37). Another study, based on the SOMAscan proteomics platform, screened three proteins (Delta-like 1, endothelial cell adhesion molecule, and mitogen-activated protein kinase 11) as candidate biomarkers for predicting the risk of DKD progression to renal failure (38). Several biomarkers have been reported to increase over time before the onset of albuminuria (39). Therefore, the discovery and identification of additional biomarkers to reliably predict renal function progression in patients with DKD are essential.

In this study, we demonstrated, for the first time, that serum and urine HMGB1 levels were significantly higher in patients with DKD than in T2DM patients without DKD and healthy controls. Serum and urine HMGB1 levels were significantly associated with known markers of renal progression. Serum HMGB1 levels were more effective than urine HMGB1 levels in predicting the occurrence of DKD. Interestingly, on the basis of RCS, we found a nonlinear relationship between serum HMGB1 levels and the progression of renal function in DKD. When the serum HMGB1 was <27 ng/ml, the risk of DKD progression was almost stable and weak. However, when the HMGB1 level was ≥27 ng/ml, the risk of DKD progression increased sharply.

In our study, both age and sex were associated with elevated serum HMGB1 levels. A preclinical study, which supports our findings, reported that HMGB1 expression varied between the sexes in kidney injury and increased more in male rats with kidney injury than in female rats (40). We also found that the expression of serum HMGB1 was associated with smoking. A recent study confirmed our finding that nicotine promoted the release and secretion of HMGB1 by enhancing cathepsin B-dependent NLRP3 inflammasome activation, which led to the disruption of endothelial permeability (41). Additionally, an interesting finding of our study was that the positive correlation between serum HMGB1 and the duration of diabetes may indicate that serum HMGB1 plays a significant role in the underlying mechanisms of diabetes-induced renal disease. As previously discussed, HMGB1 may play a more indispensable role in chronic diseases than in acute lesions (20), although extracellular HMGB1 levels can change at the moment of injury. A preclinical study also found that in the early stage after unilateral ureteral obstruction, renal tubular HMGB1 deletion had no obvious effect on renal injury, but it can significantly reduce renal interstitial fibrosis in the late/subacute stage (42). As described in our study and the literature, serum HMGB1 plays an important role in DKD, emphasizing the importance of serum HMGB1 as a biomarker for the occurrence and progression of DKD.

HMGB1 is a member of the family of high-mobility proteins with secretory and intracellular activities (43). As a DNA chaperone, autophagy regulator, and damage-associated molecular pattern (DAMPs), HMGB1 is ubiquitously expressed in almost all cell types and plays an important role in DNA repair, telomere maintenance, autophagy homeostasis, and immune regulation (44, 45). HMGB1, as a biomarker and drug target, is related to the progression of many diseases (46, 47). In kidney disease, serum HMGB1 levels are significantly elevated and show differences in different pathologies. Elevated serum HMGB1 levels are associated with renal function progression and risks of inflammation, malnutrition, and cardiovascular disease in patients with CKD (48, 49). Similarly, serum and urine HMGB1 levels have been found to be associated with disease activity and renal involvement in patients with systemic lupus erythematosus (50, 51). Interestingly, serum HMGB1 levels have also been found to be independently associated with coronary artery disease and carotid plaque susceptibility in diabetic populations (52, 53). On the basis of this evidence, we explored the association between HMGB1 and DKD in a population with diabetes. This study found, for the first time, that the serum and urine levels of HMGB1 in patients with DKD were significantly higher than those in the non-DKD diabetic and healthy control populations (2.5–10 times), and with the aggravation of DKD, serum HMGB1 levels increased significantly and was positively correlated with the serum TNFR-1 level. In summary, serum HMGB1 may reflect the regulatory response of the inflammatory state during DKD pathogenesis, and can be used as a potential marker for the prediction of DKD, which is helpful for the diagnosis and targeted treatment of DKD.

Based on the above evidence, we hypothesized several main reasons why serum HMGB1 could be used as a biomarker of DKD progression. First, HMGB1 promotes renal inflammation by recruiting immune cells and activating the nuclear factor-kB pathway, which in turn mediates the apoptosis of glomerular cells and deposition of the mesangial matrix, thereby aggravating proteinuria and glomerulosclerosis. On the other hand, circulating HMGB1 exacerbates renal fibrosis by promoting renal epithelial cell and macrophage trans-differentiation, thereby promoting renal disease progression. Hence, we hypothesized that elevated serum HMGB1 levels may reflect a persistent chronic inflammatory state in DKD. However, the detailed origin of circulating HMGB1 in DKD cannot be determined. Studies have shown that renal tubular cells and podocytes are the main sources of HMGB1 secretion in the kidneys (54). There is evidence that receptor of advanced glycation endproducts (RAGE) is the primary receptor for HMGB1, and deletion of bone marrow-derived RAGE was shown to improve renal function in a DKD mouse model (55). On the other hand, studies have shown that splenectomy can transiently reduce circulating HMGB1 levels, which in turn improves CKD (56). The current results do not explain the origin of HMGB1 in DKD in detail. This is critical for targeted therapy of DKD, and further research should be conducted to investigate this issue.

Notably, HMGB1 can be neutralized or inhibited by polypeptides, natural products, or small molecules, and exhibits potential benefits in the treatment of various diseases (57–59). Additionally, studies have shown that serum HMGB1 levels are associated with disease treatment outcomes and that high levels of HMGB1 may indicate greater sensitivity to drugs (60). Interestingly, despite increased renal tissue expression and serum HMGB1 levels in lupus nephritis, HMGB1 expression in the serum and tissues did not decrease after immunosuppressive therapy (61). These studies’ findings suggest that HMGB1 plays a complex role in the treatment of different diseases; however, the changes in HMGB1 during DKD treatment and their correlation with treatment effects remain unknown. Here, although we observed elevated levels of HMGB1 in the serum and urine of patients with DKD, we lacked follow-up information on patients after treatment. Therefore, we could not confirm a causal relationship between HMGB1 and DKD or the exact mechanism by which HMGB1 affects DKD. Therefore, future studies should focus on changes in HMGB1 levels before and after treatment, which will help in the treatment and management of DKD.

This was a preliminary study showing a correlation between elevated serum HMGB1 levels and DKD progression. However, this study has some limitations that must be addressed. First, this was a cross-sectional study, and we did not perform continuous HMGB1 measurements and long-term follow-up of the kidneys; therefore, we could not determine the causal relationship between HMGB1 and disease and the response of HMGB1 in DKD after drug treatment. Thus, a cohort study to explore whether improving serum HMGB1 levels through pharmacological targeting will affect the prognosis of DKD would be a better option to reflect the true relationship between HMGB1 and DKD and evaluate its predictive value. Second, the study population was derived from only two centers; therefore, the results should be validated in more central studies. Finally, the study population included only adults from China; thus, this association should be confirmed in other ethnic and age groups.

## 5 Conclusions

In conclusion, this is the first study to report that serum and urine HMGB1 levels are significantly increased in patients with DKD and are closely related to disease progression and renal inflammation. When the serum HMGB1 was ≥27 ng/ml, the risk of DKD progression increased sharply, suggesting that serum HMGB1 could be used as a potential biomarker for the progression of DKD. Further research on the detailed role of HMGB1 in the pathogenesis of DKD is required to guide its clinical treatment and management.

## Data availability statement

The raw data supporting the conclusions of this article will be made available by the authors, without undue reservation.

## Ethics statement

The studies involving humans were approved by Ethics Committee of Guang’anmen Hospital of the Chinese Academy of Traditional Chinese Medicine and China-Japan Friendship Hospital. The studies were conducted in accordance with the local legislation and institutional requirements. The participants provided their written informed consent to participate in this study.

## Author contributions

TL: Data curation, Formal analysis, Investigation, Software, Visualization, Writing – original draft, Writing – review & editing. HZ: Data curation, Writing – original draft. YW: Data curation, Formal Analysis, Investigation, Writing – original draft. PQ: Data curation, Methodology, Writing – original draft. YMW: Data curation, Methodology, Writing – original draft. XW: Data curation, Investigation, Writing – original draft. TZ: Data curation, Formal analysis, Project administration, Writing – original draft. LY: Data curation, Methodology, Writing – review & editing. HM: Data curation, Formal analysis, Writing – review & editing. LP: Conceptualization, Writing – review & editing. YZ: Conceptualization, Funding acquisition, Writing – review & editing. PL: Funding acquisition, Supervision, Writing – review & editing.
